# Identification and verification of the prognostic value of CUL7 in colon adenocarcinoma

**DOI:** 10.3389/fimmu.2022.1043512

**Published:** 2022-10-11

**Authors:** Chengxing Wang, Zhenyu Zhao, Yuhao Zhang, Weijun Liang, Chaorong Zhou, Weixing Lin, Yu He, Meimei Wu, Zijie Meng, Yuehua Liao, Min Li, Mariya El Akkawi, Jinglin Zhao, Yaoming He

**Affiliations:** ^1^ Department of Gastrointestinal Surgery, Jiangmen Central Hospital, Jiangmen, China; ^2^ The First Affiliated Hospital, Jinan University, Guangzhou, China; ^3^ National Drug Clinical Trial Institution, Jiangmen Central Hospital, Jiangmen, China; ^4^ Clinical Experimental Center, Jiangmen Key Laboratory of Clinical Biobanks and Translational Research, Jiangmen Central Hospital, Jiangmen, China; ^5^ Department of Pathology, Jiangmen Central Hospital, Jiangmen, China; ^6^ Department of Plastic and Aesthetic Surgery, Zhujiang hospital of Southern Medical University, Guangzhou, China

**Keywords:** CUL7, pan-cancer, COAD, prognosis, tumor immunity

## Abstract

CUL7, a gene composed of 26 exons associated with cullin 7 protein, is also an E3 ligase that is closely related to cell senescence, apoptosis, and cell transformation and also plays an important role in human cancer. However, there is no systematic pan-cancer analysis has been performed to explore its role in prognosis and immune prediction. In this study, the expression of CUL7 in colon adenocarcinoma (COAD) was investigated to determine its prognosis value. First, based on the Cancer Genome Atlas (TCGA), Genotypic-Tissue Expression Project(GTEx), Cancer Cell Line Encyclopedias(CCLE), and TISIDB database, the potential role of CUL7 in different tumors was explored. Subsequently, the expression of CUL7 in COAD was explored and verified by Immunohistochemistry (IHC). Furthermore, the mutation frequency of CUL7 in COAD was analyzed, and the prognostic value of CUL7 in COAD was discussed. In addition, the nomogram was constructed, and its prognostic value was verified by follow-up data from Jiangmen Central Hospital. Finally, PPI network analysis explored the potential biological function of CUL7 in COAD. The results show that CUL7 is upregulated in most tumors, which is significantly associated with poor survival. At the same time, CUL7 is correlated with the clinical stage and immune landscape of various tumors. In colorectal cancer, CUL7 was overexpressed in tumor tissues by IHC with a mutation frequency of about 4%. CUL7 is an independent prognostic factor for colorectal cancer. The nomogram constructed has effective predictive performance, and external databases proved the prognostic value of CUL7. In addition, PPI network analysis showed that CUL7 was closely related to FBXW8, and further pathway enrichment analysis showed that CUL7 was mainly involved in ubiquitin-mediated proteolysis. Therefore, our study provides a comprehensive understanding of the potential role of CUL7 in different tumors, and CUL7 might be a prognostic marker for COAD.

## Introduction

Cancer is widely acknowledged to be a genomic disease, and recent advancements in sequencing and informatics have cemented genomics’ standing as a cornerstone of cancer research, playing a vital role in cancer molecular research (1, 2). Research into cancer driver mutations and finer molecular subtypes is also evolving (3, 4). As more and more molecular studies of tumors from other organs are conducted, it is progressively becoming clear that some genes, like TP53, have similar effects in many cancers and that their mutations can cause basal-like breast cancer, endometrial cancer, and ovarian cancer as a result (5, 6). On the other hand, some genes, such as NOTCH, have been found to play a role in tumors of different organs, but with dissimilar effects (7–9). This leads us to the conclusion that pan-cancer analysis, which looks at similarities and differences across diverse cancer types, has developed into a potent method for acquiring new information about cancer biology (10). We mainly focused on COAD out of all the cancer types we studied since it is one of the top causes of cancer-related death globally as well as the current research being done into its treatment (11, 12). It is undeniable that the prognosis of COAD is still unsatisfactory and still plays an important role in the world’s health burden (13).

Cullin 7 (CUL7), also known as KIAA0076, p193, or p185, is a gene composed of 26 exons related to the CUL7 protein, and CUL7 is also an E3 ligase, which can promote the dissolution of the proteasome (14). In terms of cell division, Okabe et al. found that CUL7 is closely related to the proteasomal degradation of cyclin D1, which is mainly dependent on the phosphorylation of cyclin D1 residue T286 by ERK2 MAP kinase, which enables cyclin D1 ubiquitin change (15). Through the regulation of cellular protein D1, CUL7 is closely related to cellular senescence, apoptosis, and cellular transformation (16). CUL7 is found to be significantly expressed in breast, lung, hepatic, ovarian, and other malignancies and is connected to the development and incidence of several cancers (17). By mediating the degradation of HPK1, Wang and his colleagues discovered that CUL7/Fbxw8f ubiquitin ligase can play a significant role in the development of pancreatic cancer (18). Zhi et al. found that CUL7 can also be used as a biomarker for prognosis in colorectal cancer (CRC). However, even though there have been many studies involving the role of CUL7 in tumors, the complete mechanism of CUL7 remains to be studied. Therefore, research into the CUL7 gene’s aberrant expression in malignancies has significant therapeutic implications.

## Material and methods

### CUL7 expression spectrum

We retrieved RNA sequencing information, survival data, and clinicopathological characteristics linked to 33 cancer types from TCGA of the online database UCEC (https://xena.ucsc.edu). The GTEX database (http://commonfund.nih.gov/GTEx) was used to retrieve RNA sequencing information for normal tissues. From the CCLE database (portals.broadinstitute.org), expression data for each tumor cell line was extracted. The mutation MAF data and corresponding clinical information of COAD were obtained from the TCGA database. The somatic mutations of COAD patients were downloaded and visualized by the MAFTools software package in R software, and the mutation frequencies of COAD patients were displayed by a horizontal histogram.

### Survival analysis

Kaplan-Meier survival analysis was performed to investigate the differential survival outcomes between the high and low CUL7 expression groups according to the median CUL7 expression level. Univariate Cox regression models were used to determine the favorable or unfavorable prognostic value of CUL1 including overall survival (OS), disease-specific survival (DSS), disease-free interval (DFI), and progression-free interval (PFI). The R packages “survminer” and “survival” were used to carry out the KM analysis, while “survival” and “forestplot” were used to create the forestplot of Cox regression. Forest plots were created using the “Forestplot” software to show each variable (P-value, HR, and 95%CI) for both the univariate and multivariate Cox regression analyses. A nomogram was developed using the “rms” package to predict the 1-, 3-, and 5-year overall recurrence rate based on the results of multivariate Cox proportional hazards analysis. The nomogram offers graphical data for these factors, and the points assigned to each risk factor may be used to determine the prognosis risk for patients.

### Diagnostic value of CUL7

Each sample provided by TCGA was mined to select tumor stages and analyze the association of the remaining CUL7 expression, which was visualized using “ggplot2”. To assess the diagnostic precision of CUL7, a ROC curve analysis based on sensitivity and specificity was performed using the “pROC” tool. Area under the curve (AUC) values ranged from 1.0 (perfect diagnosis) to 0.5. (no diagnostic value).

### The role of CUL7 was analyzed by TISIDB

TISIDB is a web-based platform for tumor immunoassays that comprises a wide variety of heterogeneous data types from the TCGA database (http://cis.hku.hk/TISIDB/index.php). We used the TISIDB database to analyze the correlation between CUL7 expression and immune and molecular subtypes. Small lift charts were used to show how the analysis of CUL7 expression relates to immune and molecular subtypes in human cancers.

### Correlation analysis of CUL7 expression in TME

The environment in which tumor cells develop and survive is known as the tumor microenvironment (TME). Stromal cells, immune cells that surround the tumor cells, and the actual tumor cells are among their many constituents. The quantity of stromal cells and immune cells in the tumor microenvironment affects the growth and development of cancer cells. StromalScore, ImmuneScore, and ESTIMATEScore, which combines ImmuneScore and StromalScore, are all computed using the R package “ESTIMATE”. After that, we examined the relationship between CUL7 and the stromal and immunological scores using R’s Spearman correlation analysis.

### Correlation analysis of CUL7 expression with tumor mutation burden and microsatellite instability

For each tumor sample, Spearman’s rank correlation coefficient was utilized to compute TMB independently. TMB is a biomarker that reflects a mutation in tumor cells. When repeating units are inserted or deleted, the length of the microsatellite changes in comparison to that of the surrounding normal tissue. This is known as microsatellite instability (MSI). To examine the relationship between CUL7, MSI, and TMB, the Spearman rank correlation coefficient was employed.

### Correlation analysis of CUL7 expression with immune infiltrating cells

To explore the correlation between CUL7 and immune cells, we used “immunedeconV”, which includes several recent algorithms, such as TIMER, xCell, MCP-counter, EPIC, and QUANTIseq. R software V4.0.3 was used for statistical analysis, and the rank-sum test was used to detect the differences between the two groups of data. P-value <0.05 was considered statistically significant.

### Correlation analysis between the expression of CUL7 expression and some genes

The chemical change of DNA known as DNA methylation interacts with histone alterations to affect gene activity. M6A is a prominent RNA modification type that is essential for the development of cancer. Programmed death receptors and their ligands are immune checkpoints. On immune cells, they are a cluster of chemicals that are expressed. Cancer is one of many illnesses that is influenced by immune checkpoint molecule dysfunction and abnormal expression. The substances that activate immune checkpoints, which suppress the immune function of the T-cells, are analyzed by tumor cells, allowing them to survive. Copper may provide a vulnerability in the fight against cancer because of its critical involvement in the genesis, severity, and course of the disease. The correlation between CUL7 and the genes for immune stimulator and chemokine receptor proteins was also examined. We used the R package “RColorBrewer” to map the correlations between CUL7 expression and DNA methylated transferase, m6A, immune stimulants, chemokines, and copper death-related genes, as demonstrated by heat maps.

### PPI network construction and functional enrichment

GeneMANIA (www.genemania.org) was used to construct gene-gene interaction networks. And perform GO/KEGG analysis.

### Tissue samples

100 pairs of COAD tissues and corresponding peritumoral normal tissues from COAD patients who had surgical resection at Jiangmen Central Hospital in Guangdong Province, China, between 2016 and 2017 were gathered for this study. None of the patients received radiotherapy, chemotherapy, or immunotherapy before the operation. Patients and volunteers both signed informed consent forms. The Jiangmen Central Hospital’s Medical Ethics Committee gave its approval to all operations (decision no. JXY202228).

### Immunohistochemistry

The test procedure was based on our earlier publication (19). Sections of COAD tissues were deparaffinized and rehydrated. To block endogenous peroxidase activity, the antigen was retrieved by submersion in citrate buffer (pH 6.0) for 15 minutes at 95°C before incubation with 0.3% hydrogen peroxide for 15 min at room temperature. Sections were treated with phosphate-buffered saline (PBS) rinsing and 5% normal goat serum (Thermo Fisher Scientific, Inc. USA) blocking for 30 minutes at room temperature before being treated with a primary anti-CUL7 antibody (1:100; Abcam, USA) and incubated overnight at 4°C. All sections were subjected to the peroxidase-anti peroxidase detection technique before being counterstained, dried off, and mounted on a coverslip at room temperature. Yellow particles in the cytoplasm and/or nucleus were used to estimate the proportion of colorectal cells that were positive. The strength of the staining was graded as either negative (–), weakly positive (+), medium (+++), or very positive (+++). The intensity score, which ranges from 0 to 3, was multiplied by the proportion of positive cells, which ranges from 0 to 300, to obtain the H-score. The H-score was computed by two skilled pathologists using a double-blind method.

### Statistical analysis

All analyses were performed by R software 4.0.3. By using the Wilcoxon rank sum test, CUL7 in tumor tissues and normal samples were compared. The Pearson correlation test was used to determine associations between the expression of CUL7 and several targets, such as TMB, MSI, immunological checkpoint genes, M6A genes, copper death genes, DNA methylation genes, immune stimulators, chemokines, and immune infiltrating cells. R values less than 0.05 were deemed significant. “ggplot2”, “ggpubr”, “limma”, “survival”, “survminer”, “fmsb”, “ggExtra”, “clusterProfiler”, “ESTIMATE”, “RColorBrewer”, “enrichplot”, and “forestplot” are some of the R programs that were utilized for the analysis.The relationship between CUL7 expression and the clinicopathological features was analyzed using the chi-square test. Numerical data were expressed as the mean ± SD. The log-rank test was used to compare the results of the Kaplan-Meier method-based survival analysis. Cox regression, both univariate and multivariate, was used to assess the variables. Statistics were judged significant at a P-value < 0.05.

## Results

### Expression of CUL7 in pan-cancer

**Figure 1:**
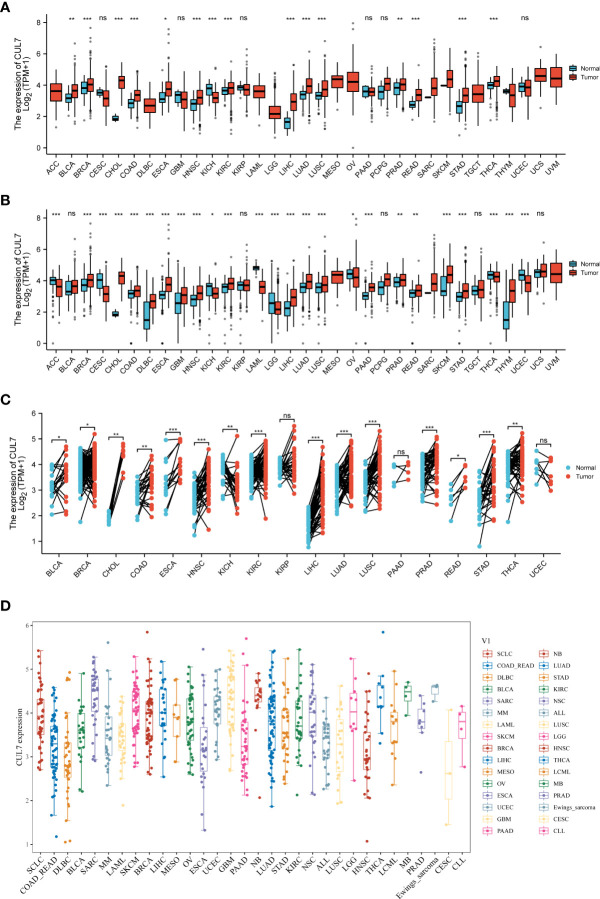
Differential expression of CUL7. **(A)** CUL7 expression in 33 tumors in TCGA. **(B)** CUL7 expression in 33 tumors in TCGA and GTEx. **(C)** Differential expression of CUL7 in paired tumors and adjacent normal tissues in TCGA. **(D)** CLU7 expression in various cancer cell lines from the CCLE database. Ns, non-significant, *P < 0.05; **p < 0.01; ***P < 0.001).

Firstly, we obtained the CUL7 expression data of 33 tumors from the TCGA database, and analyzed the CUL7 expression score, as shown in **Figure 1A**. CUL7 was highly expressed in BLCA, BRCA, CHOL, COAD, ESCA, HNSC, KIRC, LIHC, LUAD, LUSC, PRAD, READ, and STAD, and low in KICH. For further analysis, when we combined data from TCGA and GTEx databases and found that CUL7 is highly expressed in BRCA, CHOL, COAD, DLBC, ESCA, GBM, HNSC, KIRC, LIHC, LUAD, LUSC, PAAD, PRAD, READ, SKCM, STAD, and THYM. Low expression was found in ACC, CESC, KICH, LAML, LGG, OV, THCA, and UCEC (**Figure 1B**). In TCGA paired analysis, CUL7 was found to be highly expressed in BLCA, BRCA, CHOL, COAD, ESCA, HNSC, KICH, KIRC, KIRP, LIHC, LUAD, LUSC, PRAD, READ, STAD, and THCA (**Figure 1C**). We downloaded the expression of various cell lines from the CCLE database and used box plots to present the expression of CUL7 in different cell lines (**Figure 1D**).

### Prognostic value of CUL7 in pan-cancer

**Figure 2:**
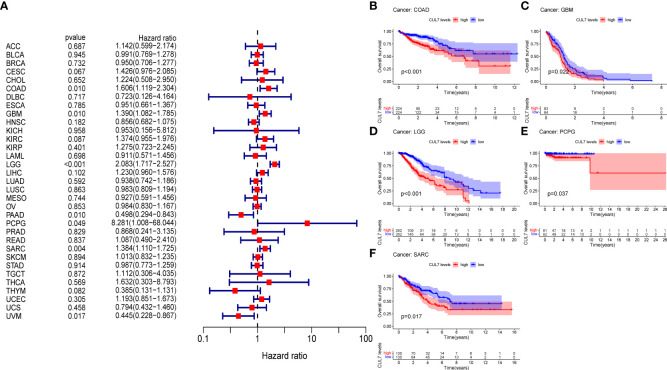
Relationship between OS and CUL7 expression. **(A)** Forest plot of univariate Cox regression analysis of OS. **(B–F)** Kaplan-Meier diagram of COAD, SARC, GBM, LGG, and PCPG.

We looked at the relationship between CUL7 expression and several survival outcomes for each cancer, including OS, DFI, PFI, and DSS, to better understand the prognostic value of CUL7 in pan-cancer. Analysis using the Cox proportional hazards model revealed that CUL7’s expression level was substantially greater than COAD (HR=1.606, P=0.01), GBM (HR=1.390, P=0.01), LGG (HR=2.083, P<0.001), PAAD (HR=0.498, P<0.001). P=0.01), PCPG (HR=8.281, P=0.049), SARC (HR=1.384, P=0.04), and UVM (HR=0.445, P=0.017). These results indicated that CUL7 was a high-risk gene in COAD, GBM, LGG, PCPG, SARC, and a low-risk gene in PAAD and UVM (**Figure 2A**). Kaplan-Meier survival analysis showed that high CUL7 expression was associated with poor OS in COAD (**Figure 2B**), GBM (**Figure 2C**), LGG (**Figure 2D**), and PCPG (**Figure 2E**), SARC (**Figure 2F**).

In addition, the analysis of DSS data (**Figure S1A**) showed that the high expression of CUL7 was significantly associated with poor prognosis in COAD (HR=1.647, P=0.029), GBM (HR=1.471, P=0.007), LGG (HR=2.832, P<0.001), PCPG (HR=16.418, P<0.001), and COAD (HR=1.647, P=0.029). P=0.033), SARC (HR=1.355, P=0.014) and THCA (HR=30.871, P=0.049) patients, while CUL7 low expression was associated with poor prognosis in PAAD (HR=0.497, P=0.021) and UVM (HR=0.501, P=0.021). P=0.044) patients. Kaplan-Meier survival studies showed that high CUL7 expression was associated with poor DSS in COAD (**Figure S1B**), GBM (**Figure S1C**), SARC (**Figure S1F**), LGG (**Figure S1D**), and PCPG (**Figure S1E**). We further analyzed the relationship between gene expression and DFI and PFI.

In DFI, the high expression of CUL7 was associated with the poor prognosis of CESC (HR=2.237, P=0.010), COAD (HR=3.842, P<0.001), and PRAD (HR=3.005, P=0.014) patients (**Figure S2A**). Kaplan-Meier survival studies showed that high CUL7 expression was associated with poorer DFI in COAD (**Figure S2B**).

In PFI, the high expression of CUL7 was significantly associated with the poor prognosis of CESC (HR=1.563, P=0.017), COAD (HR=1.631, P=0.003), LGG (HR=2.227, P<0.001), LIHC (HR=1.302, P=0.015), PRAD (HR=1.752, HR=1.752), COAD (HR=1.631, P=0.003). P=0.021) and TGCA (HR=2.929, P=0.029) patients, while the low expression of CUL7 in PAAD (HR=0.435, P=0.002) patients was associated with poor prognosis (**Figure S3A**). Kaplan-Meier survival studies showed that high CUL7 expression was associated with poor PFI in COAD (**Figure S3B)**, LGG (**Figure S3C**), LIHC (**Figure S3D**), and THYM (**Figure S3E)**, whereas low CUL7 expression was associated with poor PFI.

### Diagnostic value of CUL7 in cancer

**Figure 3:**
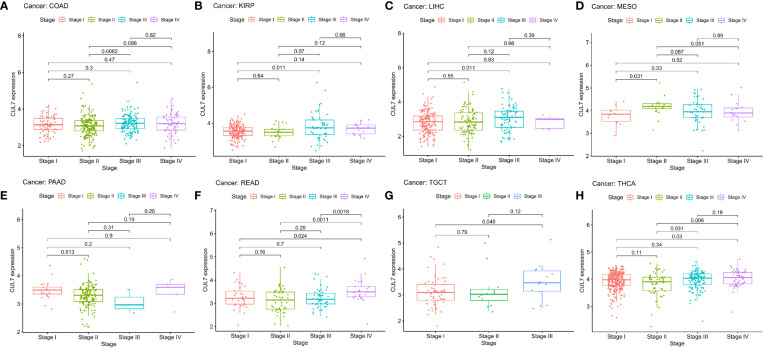
Correlation between CUL expression and tumor pathological grades (Stage I, II, III, IV). **(A)** COAD, **(B)** KIRP, **(C)** LIHC, **(D)** MESO, **(E)** PAAD, **(F)** READ, **(G)** TGCT, **(H)** THCA.

In the correlation examination of tumor stage based on TCGA, the results showed that the expression of CUL7 in stage III was increased in COAD, KIRP, LIHC, MESO, TGCT, and THCA, stage II was increased in PAAD, and stage IV was increased in READ. This implies that CUL7 could be useful clinically for the detection of malignancy (**Figure 3**). The receiver operating characteristic (ROC) curve was used to assess the diagnostic accuracy of the gene features. Different AUC cutoff values were considered to indicate high diagnostic accuracy (AUC: 0.9-1.0), relative diagnostic accuracy (AUC: 0.7-0.9), or low diagnostic accuracy (AUC: 0.5-07). The results showed that the diagnosis accuracy of CHOL and LIHC were high while that of BLCA, CESC, COAD, DLBC, ESCA, HNSC, KICH, KIRC, LUAD, LUSC, PAAD, READ, SKCM, STAD, THCA, and THYM were relative. ACC, BRCA, GBM, and PRAD had low diagnostic accuracy (**Figure S4**).

### Correlation between CUL7 and different immune cells in pan-cancer

To analyze the correlation between CUL7 and immune cells, different algorithms were used. The correlation between the degree of immune cell infiltration and CUL7 expression was significant for the majority of cancer types. The results of the MCPCOUNTER (**Figure S5A**) algorithm showed that Neutrophil was correlated with 22 kinds of cancer, and endothelial cells with 20, whereas the results of the QUANTISE1 algorithm (**Figure S5B**) showed that NK cells were correlated with 22 kinds of cancer. XCELL algorithm(**Figure S5C**) found that Myeloid dendritic cell activated was correlated with 22 kinds of cancer, while Macrophage M1 was correlated with 21, Plasmacytoid dendritic cell with 10, and Macrophage M2, Macrophage and Myeloid dendritic cells were associated with 19 types of cancer.

### Correlation of CUL7 with TMB and MSI

**Figure 4:**
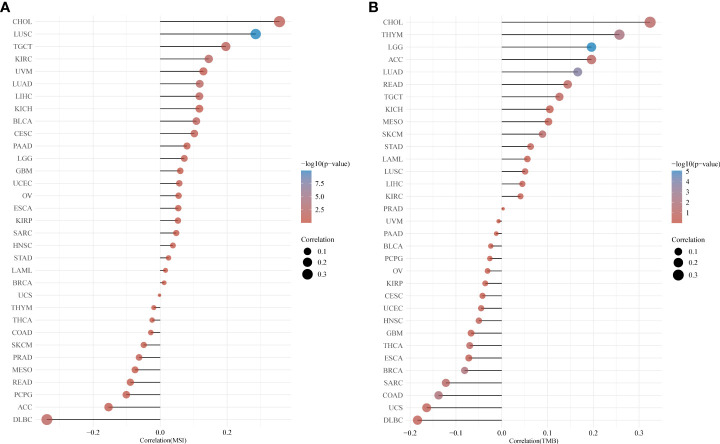
Relationship between CUL7 and TMB and MSI based on TCGA database. **(A)** Correlation between CUL7 and TMB. **(B)** Correlation between CUL7 and MSI.

TMB is a quantifiable biomarker used to reflect the number of mutations in cancer cells. Each tumor sample’s TMB was determined using the Spearman rank correlation coefficient, and the association between gene expression and TMB was examined (**Figure 4A**). CUL7 gene expression level was significantly positively correlated with LGG, LUAD, and THYM. However, it was negatively correlated with BRCA and COAD. Spearman rank correlation coefficient was used to analyze the correlation between CUL7 expression and MSI (**Figure 4B**). The results were as follows: CUL7 expression level was significantly positively correlated with BLCA, CHOL, KIRC, LIHC, LUAD, LUSC, and TGCT, and negatively correlated with DLBC.

### Relationship between CUL7 and tumor microenvironment

To explore the association of CUL7 with the TME of immune cells, stromal cells, and tumor cells, ESTIMATE R-package was used to determine immune and stromal scores for each cancer type. CUL7 expression was negatively correlated in BLCA, BRCA, KIRC, KIRP, LAML, LIHC, LUAD, LUSC, MESO, OV, PCPG, PRAD, SARC, SKCM, STAD, TGCT, THYM, and UCEC (**Figure S6**). CUL7 expression was negatively correlated with the Stromal score in BLCA, BRCA, KIRC, LAML, LUAD, MESO, PCPG, PRAD, and SKCM, and positively correlated with the stromal score in NSC, LGG, TGCT, and THYM (**Figure S7**).

### Correlation between CUL7 and immune and molecular subtypes

The TIMIB database was used to classify the correlation between CUL7 expression and immune subtypes and molecular subtypes. To examine and explain the mechanism of CUL7, the violin map with a significant correlation between CUL7 expression and immunological subtypes and molecular subtypes was chosen. Among the immune subtypes (**Figure S8**), CUL7 expression was significantly correlated with BLCA (P= 6.74E-04), BRCA (P= 2.77E-06), COAD (P= 5.51E-08), ESCA (P= 5E-02), GBM (P= 2E-02), HNSC (P= 2.58E-04), KIRP (P= 6.53E-03), L GG (P = 7.9E-06), LUSC (P = 1.5E-02), PRAD (P = 5.32E-03), READ (P = 1E-02), SARC (P = 5.58E-05), SKCM (P = 2.21E-03), STAD (P = 9.63E-03), TGCT (P =2).87E-09) and UCEC (P = 2.78E-02). Among the molecular subtypes (**Figure S9**), CUL7 expression was correlated with BRCA (P= 1.31E-03)/COAD (P= 8.27E-03)/GBM (P= 1.1E-03)/HNSC (P= 6.23E-04)/KIRP (P= 5.46E-04)/LGG (P= 1.05E-40)/OV (P= 7.96E-03)/PCPG (P= 7.87E-03)/PRAD (P= 1.6E-07)/SKCM (P= 2.6E-02)/STAD (P= 1.75E-03)/UCEC (P= 1.54E-10).

### CUL7 expression in COAD and Somatic mutation

**Figure 5:**
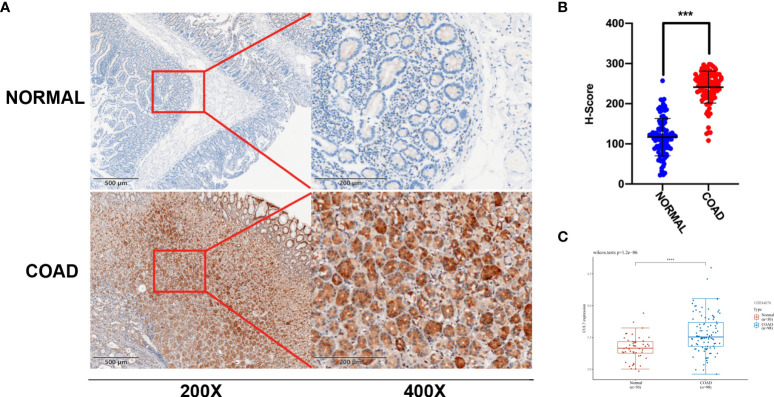
Differential expression of CUL7 **(A)** Immunohistochemical (IHC) staining of non-neoplastic muscle tissues and matched muscle cancer tissue sections. (n = 100; Scale bars, 500 μm and 200 μm). **(B)** The Scatter plot indicated the H-score of Gankyrin IHC staining intensity. **(C)** CUL7 expression in GSE44076. Ns, non-significant, ***P < 0.001; ****P < 0.0001).

In the above prognostic analysis, we found that the high expression of CUL7 in OS/DSS/DFI/PFI had a poor prognosis in COAD. We further analyzed GES44076 (**Figure 5C**), and the results showed that CUL7 is highly expressed in COAD. We selected cancer tissues and adjacent normal tissues from 100 patients with rectal cancer in Jiangmen Central Hospital for IHC analysis and found that cul7 was highly expressed in cancer tissues (**Figures 5A, B**).

**Figure 6:**
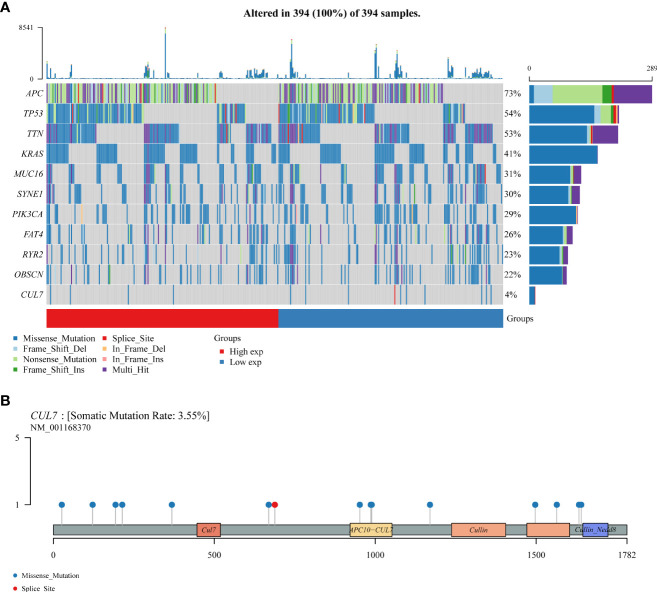
The somatic mutation landscape of CUL in COAD. **(A)** Oncoplot showing the somatic landscape of the COAD tumor cohort. Genes are sorted according to mutation frequency. **(B)** CUL7 gene mutation distribution map.

Somatic mutations in the COAD cohort showed that APC (73%), TP53 (54%), TTN (53%), and CUL7 (4%) had higher mutation rates (**Figure 6A**). CUL7 mutation distribution is shown in **Figure 6B**.

### CUL7-related central genes

**Figure 7:**
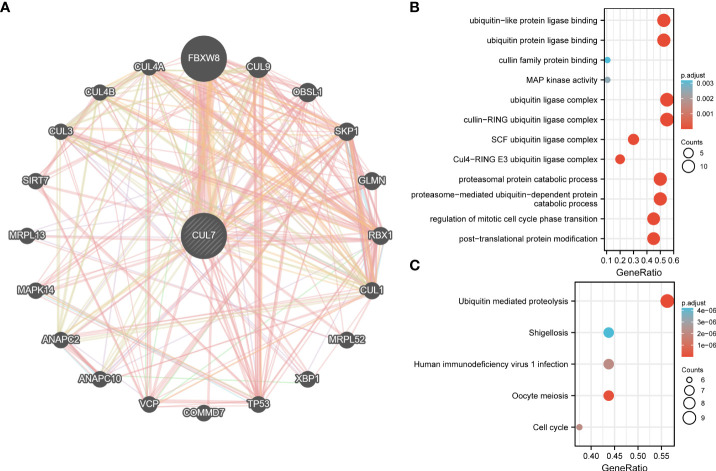
PPI network and functional enrichment analysis of CUL7. **(A)** PPI network of CUL7. **(B)** GO analysis of related genes. **(C)** KEGG analysis of related genes.

A PPI network with 20 nodes was built in the Genemania database to comprehend the association between CUL7 expression in COAD (**Figure 7A**). By way of physical interactions, co-expression, prediction, colocalization, genetic interactions, pathways, and common protein domains, nodes in the network demonstrated that certain genes were linked to CUL7. The gene most significantly associated with CUL7 is FBXW8. At the same time, 20 genes were analyzed by GO/KEGG. GO results analysis (**Figure 7B**) showed that in BP, mainly enriched to proteasome-mediated ubiquitin-dependent protein catabolic process and Proteasomal protein catabolic process. In CC, Mainly enriched to Cullin-ring Ubiquitin ligase complex and Ubiquitin ligase complex. In MF, It was mainly enriched to ubiquitin protein ligase binding and ubiquitin-like protein ligase binding. KEGG analysis (**Figure 7C**) showed that Ubiquitin mediated proteolysis, Oocyte Meiosis, Human immunodeficiency virus 1 infection, and Cell cycle were mainly enriched.

### Prognosis of CUL7 in COAD

**Figure 8:**
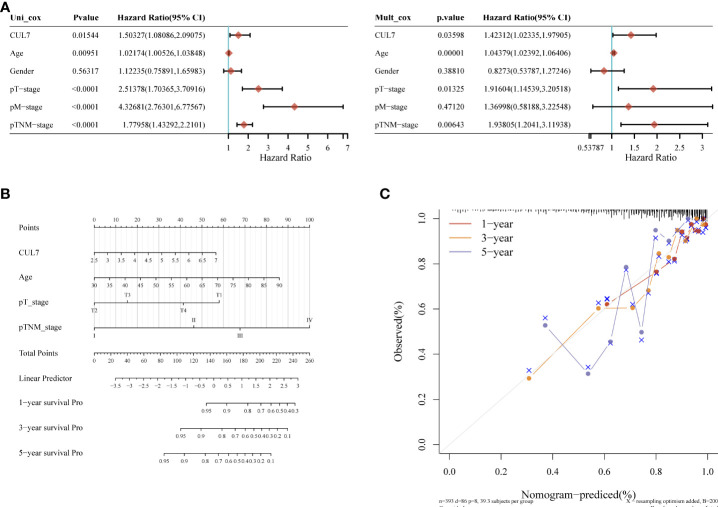
Kaplan-Meier overall survival (OS) analysis of COAD patients with high and low expression levels of CUL7. **(A)** mono-factor and multi-factor analysis. **(B)** Nomogram of multi-factor analysis results. **(C)** Calibration diagram.

**Figure 9:**
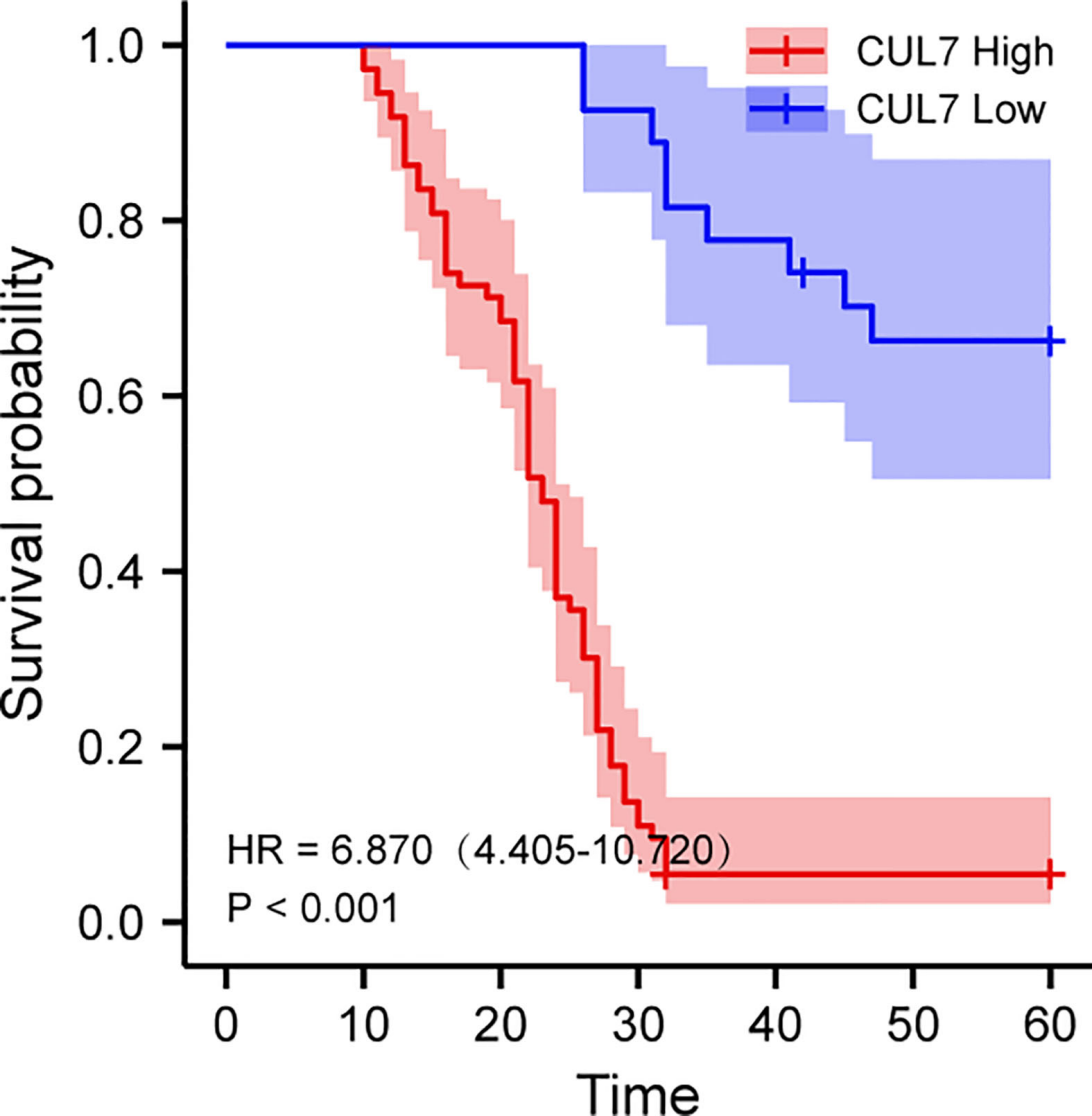
Kaplan-Meier overall survival (OS) analysis of COAD patients with high and low expression levels of CUL7.

We conducted a prognosis analysis of CUL7 in COAD, and constructed prognosis models for OS, DFS, and PFS respectively. According to the results of OS analysis (**Figure 8**), CUL7, Age, T stage, and TNM stage were found to be independent factors combined with Univariate and multiple factors (**Figure 8A**). Nomogram was constructed to show the correlation between CUL7 expression and 1-, 3-, and 5-year survival probability (**Figures 8B, C**). The results indicated that higher CUL7 expression predicted lower survival probability. In DFS analysis (**Figure S10**), CUL7, Age, and T stage were found to be independent factors, and a nomogram was constructed to show the correlation between CUL7 expression and 1-, 3-, and 5-year survival probability. The results indicated that higher expression of CUL7 predicted lower survival probability. In the PFS analysis (**Figure S11**), CUL7, T stage, and TNM stage were found to be independent factors, and the nomogram was constructed to show the correlation between CUL7 expression and 1-, 3- and 5-year survival probability, suggesting that higher expression of CUL7 predicted lower survival probability. Based on the above analysis results, we speculate that CUL7 has a good predictive role in COAD. We collected the clinical data of 100 rectal cancer patients and performed OS analysis. It was discovered that a worse prognosis was linked to increased CUL7 expression (**Figure 9**). Next, the risk factors of OS in 100 rectal cancer patients were analyzed by univariate and multivariate Cox regression analysis. TNM stage (P = 0.006) and high CUL7 expression (P = 0.001) were risk variables linked to poor outcomes for individuals with rectal cancer, according to multivariate Cox regression analysis (**Table 1**).

**Table 1:** Cox regression analysis of five years overall survival in 100 COAD patients.

Univariate cox regression analysis	Overall survival		
	Hazard Ratio	95% CI	P-value
Age (≥55years vs <55 years)	1.004	0.638-1.581	0.985
Gender (Male vs Female)	1.137	0.723-1.786	0.578
Smoke (Yes vs No)	1.257	0.803-1.968	0.317
Drink (Yes vs No)	0.910	0.579-1.430	0.683
CEA (≥ 5 ng/ml vs < 5 ng/ml)	7.208	3.833-13.555	< 0.001
Tumor size (≥ 5 cm vs < 5 cm)	0.826	0.529-1.289	0.400
Tumor location (right vs left)	0.872	0.557-1.365	0.549
TNM stage (III + IV vs I + II)	4.558	2.638-7.876	< 0.001
Family history (Yes vs No)	1.554	0.855-2.824	0.148
Lymph node metastasis (Positive vs Negative)	8.150	4.035-16.462	< 0.001
CUL7 expression (High vs Low)	9.020	4.347-18.716	< 0.001
Multivariate cox regression analysis	Overall survival		
	Hazard Ratio	95% CI	P-value
CEA (≥ 5 ng/ml vs < 5 ng/ml)	1.150	0.320-4.126	0.831
TNM stage (III + IV vs I + II)	2.649	1.330-5.277	0.006
Lymph node metastasis (Positive vs Negative)	1.333	0.329-5.403	0.687
CUL7 expression (High vs Low)	5.302	1.946-14.450	0.001

## Discussion

Colon adenocarcinoma (COAD) is the second or third leading cause of cancer death in adults over 20 years of age worldwide, and is prone to recurrence and metastasis, with a 5-year survival rate of less than 15% (20). Although anti-COAD treatment methods are diverse, including surgery, chemotherapy, local ablation therapy, targeted therapy, immunotherapy, and palliative care (21), the strong invasiveness, high metastases, and high recurrence rate make the therapeutic effect of COAD not satisfactory. The overall survival rate has not been significantly improved (22). Early diagnosis and intervention are important to reduce disease morbidity and improve prognosis. With the development of precision medicine, the search for predictive biomarkers is welcomed by oncology researchers (23). Biomarker studies on COAD are increasing year by year. For example, Sun et al. found in a mouse model that miR-195-5p can be used as a predictive biomarker to judge the outcome of colorectal cancer (CRC) patients with certain accuracy (24). Additionally, IRS and miR-21 were discovered by Schetter and his team to be independent predictors of colon cancer (25). It is undeniable that colon cancer and rectal cancer are often clinically linked and studied as CRC. Independent biological predictors of COAD itself still need to be explored. Tumor cells exist in a dynamic environment and interact with tumors, called the tumor microenvironment (TME), which plays an important role in the growth, invasion, metastasis, and escape of tumor cells (26). The research on the relationship between TME and tumor cells has become a core issue and a hot spot in anti-tumor therapy (27). Combining molecular and TME research may provide new directions for improving cancer treatment.

CUL7, a new gene reported in 2013, is closely related to the occurrence of hepatocellular carcinoma (HCC) (28). There are currently two scenarios for the mechanism of action of CUL7 in cancer. The vast majority of studies have shown that the high expression of CUL7 mainly affects the E3 ubiquitin pathway by forming Cullin (CUL)-RING E3 ubiquitin ligase (CRL), inhibiting the expression of cyclins, thereby promoting the proliferation of cancer cells and reducing apoptosis (17, 29, 30). However, Jung et al. pointed out that CUL7 may also directly interact with the P53 pathway to affect the occurrence and development of cancer (31). Some studies have also pointed out that the anticancer drug 3,3’-diindolylmethane (DIM) can interact with the TME of gastric cancer cells through E3 ubiquitin ligase (32). A review investigating the role of ubiquitination in tumorigenesis indicated that P53 ubiquitination significantly modulates the TME (33). Unfortunately, neither the E3 ubiquitin ligase nor the p53 pathway has been reported for their role and CUL7 in COAD patients.

Given the above characteristics, we conducted a comprehensive and in-depth evaluation system to analyze the correlation of CUL7 expression with cancer progression and tumor stage through Kaplan-Meier survival analysis, combined with the study of changes in immune cells. At the same time, we also studied the interaction between CUL7 and drugs to provide new therapeutic targets for prediction, diagnosis, and clinical treatment.

In the TCGA dataset of this study, the expression of CUL7 was significantly increased in most tumor tissues including COAD compared with normal tissues, while the expression was decreased in some cancers such as ACC, KICH, and LAML, suggesting CUL7 may have oncogenic or tumor suppressor effects. Subsequent analyses of the HPA dataset showed similar results. Kaplan-Meier (KM) survival curves showed that higher CUL7 was associated with worse overall survival (OS) and poor prognosis, in particular, disease-specific survival (DSS) and disease-free interval (DFI) in the COAD subgroup) were lower, suggesting that abnormal excess CUL7 is a risk factor for COAD and may be an early predictor of cancer.

The quantity of somatic mutations per megabase in the tumor genome sequence is known as tumor mutational burden (TMB), and TMB prediction helps identify patients who may benefit from immunotherapy (34). Numerous studies have demonstrated that TMB can be used as a biological predictor to predict the response of cancer patients to immune checkpoint inhibitors (ICIs), and the regulation of TMB is significantly associated with improved survival after ICI response (35–38). In COAD, the regulation of TMB has also been shown to be significantly associated with ICI response (39). Microsatellite instability (MSI) refers to a hypervariable phenotype in which tumor DNA mismatches lead to changes in the length of microsatellite (MS) sequences (40). As predictors also associated with ICI response, Long et al. analyzed 32 tumor types and MSI sequences including COAD, bladder urothelial carcinoma (BLCA), ovarian carcinoma (OV), and rectal adenocarcinoma (READ), among others. Yes, 25 tumors were found to be consistent with MSI (41). In our study, the expression of CUL7 was negatively correlated with TMB and MSI. Higher CUL7 expression was associated with lower levels of TMB and MSI, both of which are very sensitive to ICI inhibitors. Therefore, this will provide a potential therapeutic target for clinical treatment.

In addition to this, we also investigated the correlation of CUL7 expression with TME. The results showed a contradictory relationship between different tumors and a negative trend between CUL7 expression and TME score composed of the stromal score, immune score, and estimated score in COAD patients. Our study showed that the expression of CUL7 was negatively correlated with immune factors such as B cell population (including B cell, B cell plasma, B cell naïve, and B cell memory), plasmacytoid dendritic cell, and NK cell. Although there is no other literature supporting the negative correlation between CUL7 and the above immune factors, we found that CUL7 and CUL3 have similar roles through the co-expression network. Studies have shown that high expression of CUL3 is associated with the increase of E3 ubiquitin ligase or inhibition of the p53 pathway, and they are negatively correlated with the effects of B cells, plasmacytoid dendritic cells, and NK cells (42–47). It suggests that our findings have a certain credibility. In our study, according to the enrichment analysis, the high expression of CUL7 in COAD is mainly related to the decrease of cyclin caused by the action of E3 ubiquitin ligase, which is consistent with the mechanism of CUL7 in tumors mentioned above.

However, this study still has some limitations. First, although bioinformatics analysis has provided us with some important insights into CUL7 in malignancies, we have also validated the cancer-promoting role of CUL7 in COAD by molecular biology approaches, further *in vitro* or *in vivo* biological experiments to validate our results and improve treatment outcomes. In addition, the information we investigate and integrate comes from databases, and there may be data biases that require additional model validation experiments to confirm.

Overall, our findings reveal a critical involvement of CUL7 in tumorigenesis and metastasis. CUL7 is considered a potential new target for cancer therapy because they are upregulated in a variety of cancers. Our findings suggest that high expression of CUL7 is associated with poor survival and early clinical stage. At the same time, we found that CUL7 was highly expressed in COAD through IHC, and the prognosis was poor in COAD. Expression was negatively correlated with TMB and MSI, and changes in immune cells and signaling pathways brought potential therapeutic targets for clinical treatment. Future prospective studies focusing on CUL7 expression and tumor immune milieu will help to provide conclusive answers, allowing the development of immune-based anticancer therapies.

## Data availability statement

The original contributions presented in the study are included in the article/**Supplementary Material**. Further inquiries can be directed to the corresponding authors.

## Ethics statement 

The studies involving human participants were reviewed and approved by The Jiangmen Central Hospital’s Institutional Research Ethics Committee. The patients/participants provided their written informed consent to participate in this study.

## Author contributions

CW, ZZ, ME, and YMH designed the study. CW, ZZ, and YZ wrote the manuscript. YZ, WJL, CZ, WXL, YH, MW, ZM, YL, ML, and ME contributed to the collection of the data. ME and JZ contributed to the critical revision of the manuscript. All authors contributed to the article and approved the submitted version.

## Funding

This study was supported by grants from Guangdong Administration of Traditional Chinese Medicine Research Foundation (no.20221433), Guangdong Basic and Applied Basic Research Foundation (no.2021A1515220175), Guangdong Medical Research Foundation (no.B2021057), and Jiangmen Planned Project of Science and Technology (no. 2021030103670007327). The funders had no role in the study design, data collection, analysis, decision to publish, or preparation of the manuscript.

## Acknowledgments

All authors would like to express their sincere gratitude to the participants who provided public database data analysis and colon tissue samples for this study.

## Conflict of interest

The authors declare that the research was conducted in the absence of any commercial or financial relationships that could be construed as a potential conflict of interest.

## Publisher’s note

All claims expressed in this article are solely those of the authors and do not necessarily represent those of their affiliated organizations, or those of the publisher, the editors and the reviewers. Any product that may be evaluated in this article, or claim that may be made by its manufacturer, is not guaranteed or endorsed by the publisher.
