# Supplementary material for: Identification and verification of the prognostic value of CUL7 in colon adenocarcinoma
Source: Front Immunol. 2022 Oct 11;13:1043512. doi: 10.3389/fimmu.2022.1043512 (PMC9592904; doi:10.3389/fimmu.2022.1043512)
Supplement: Supplementary file 1 [file DataSheet_1.docx]

Supplementary Material

## Supplementary Figures


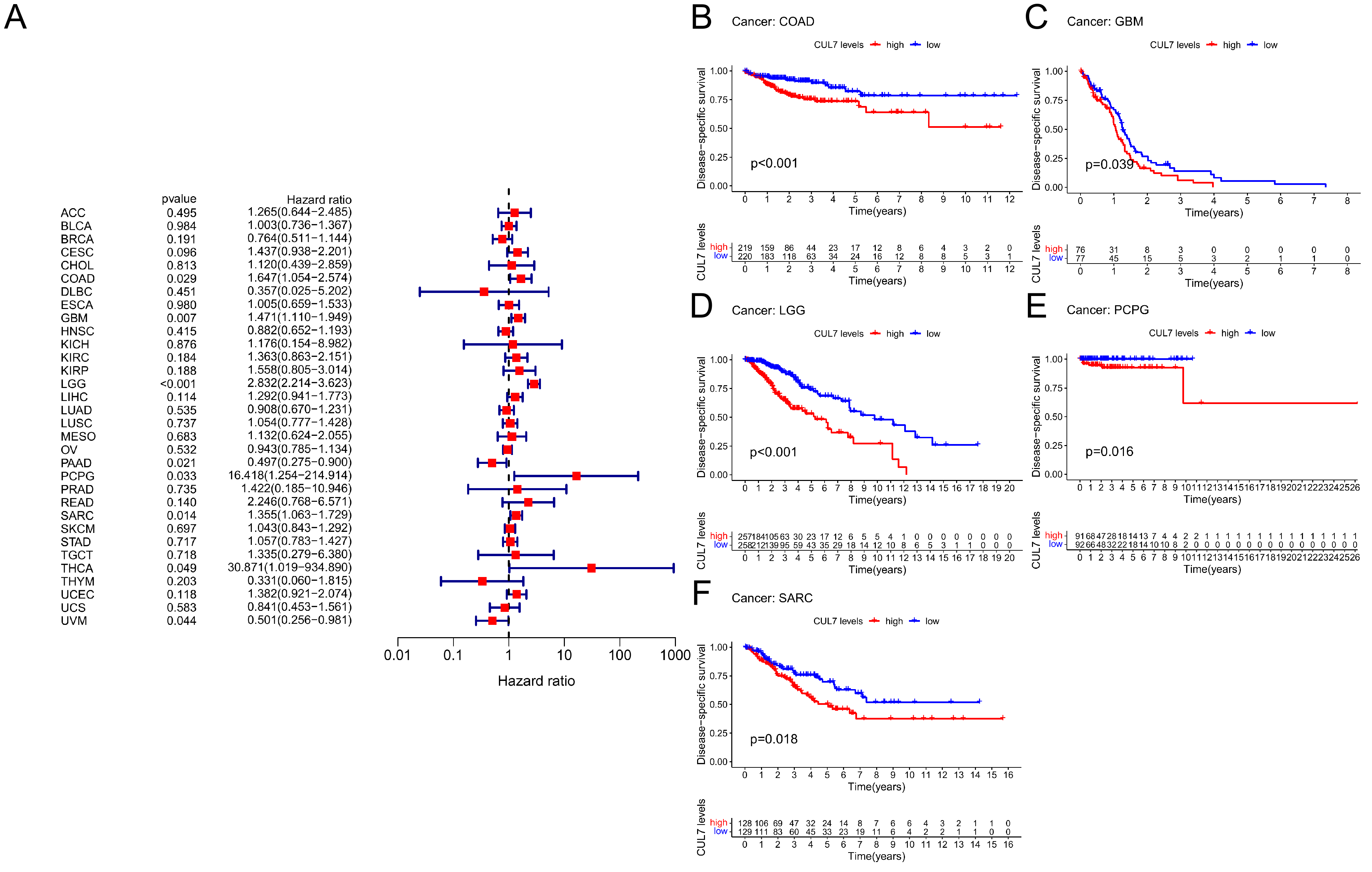


**Supplementary Figure 1.** Relationship between DSS and CUL7 expression. (A) Forest plot of univariate Cox regression analysis of DD. (B-F) Kaplan-Meier diagram of COAD, GBM, LGG, PCPG, SARC.


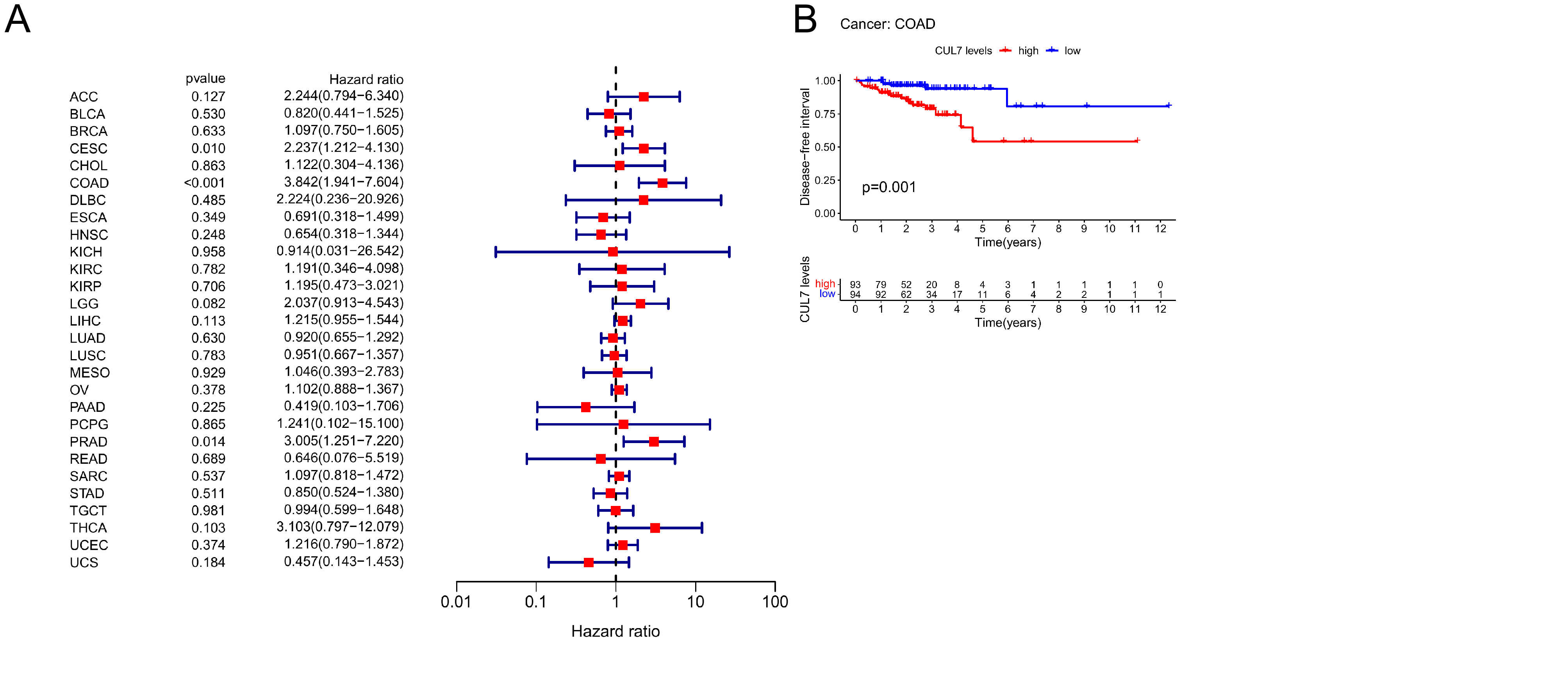


**Supplementary Figure 2.** Relationship between DFI and CUL7 expression. (A) Forest plot of univariate Cox regression analysis of DD. (B) Kaplan-Meier diagram of COAD.
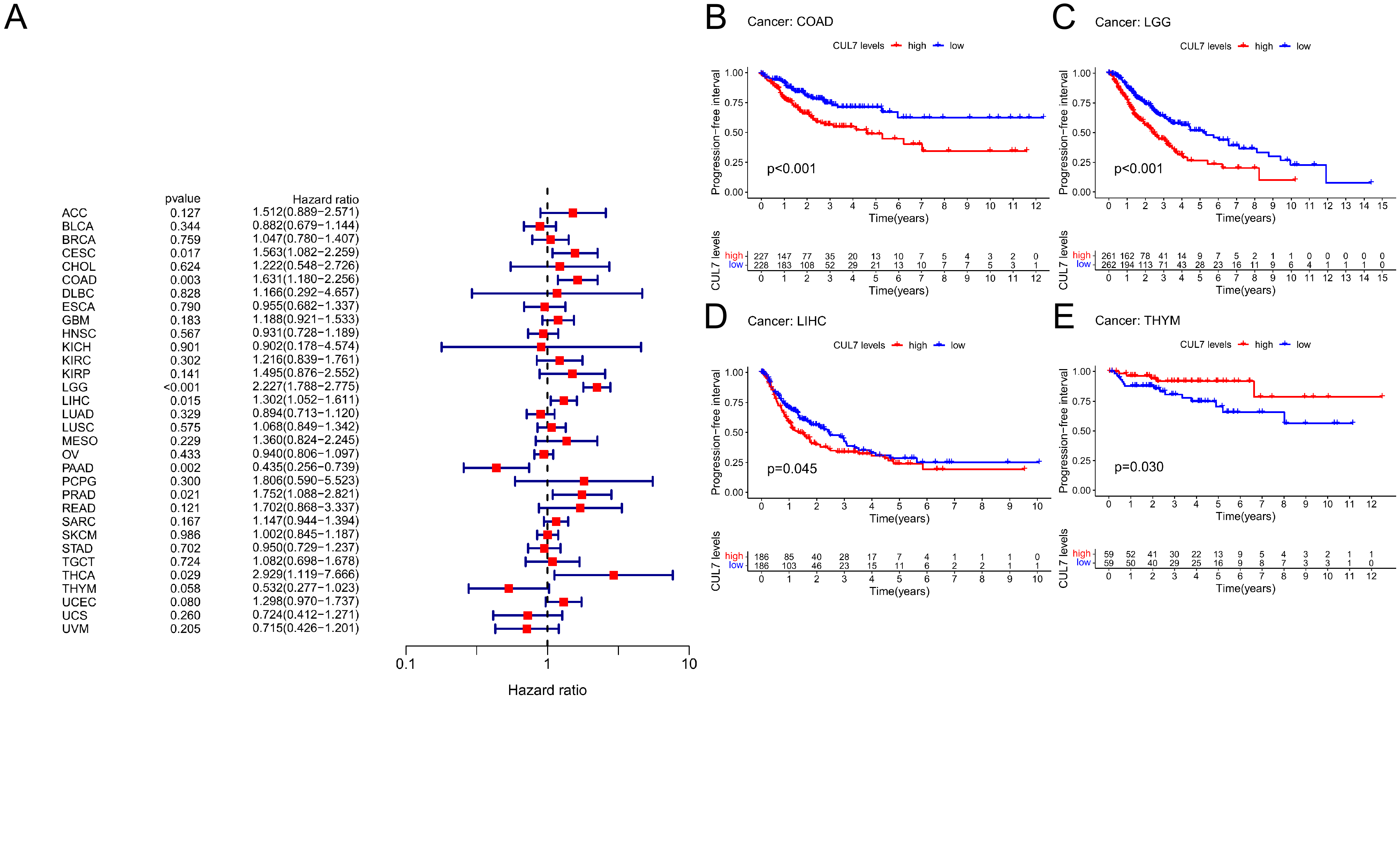


**Supplementary Figure 3.** Relationship between PFI and CUL7 expression. (A) Forest plot of univariate Cox regression analysis of DD. (B-F) Kaplan-Meier diagram of COAD, LGG, LIHC, THYM.


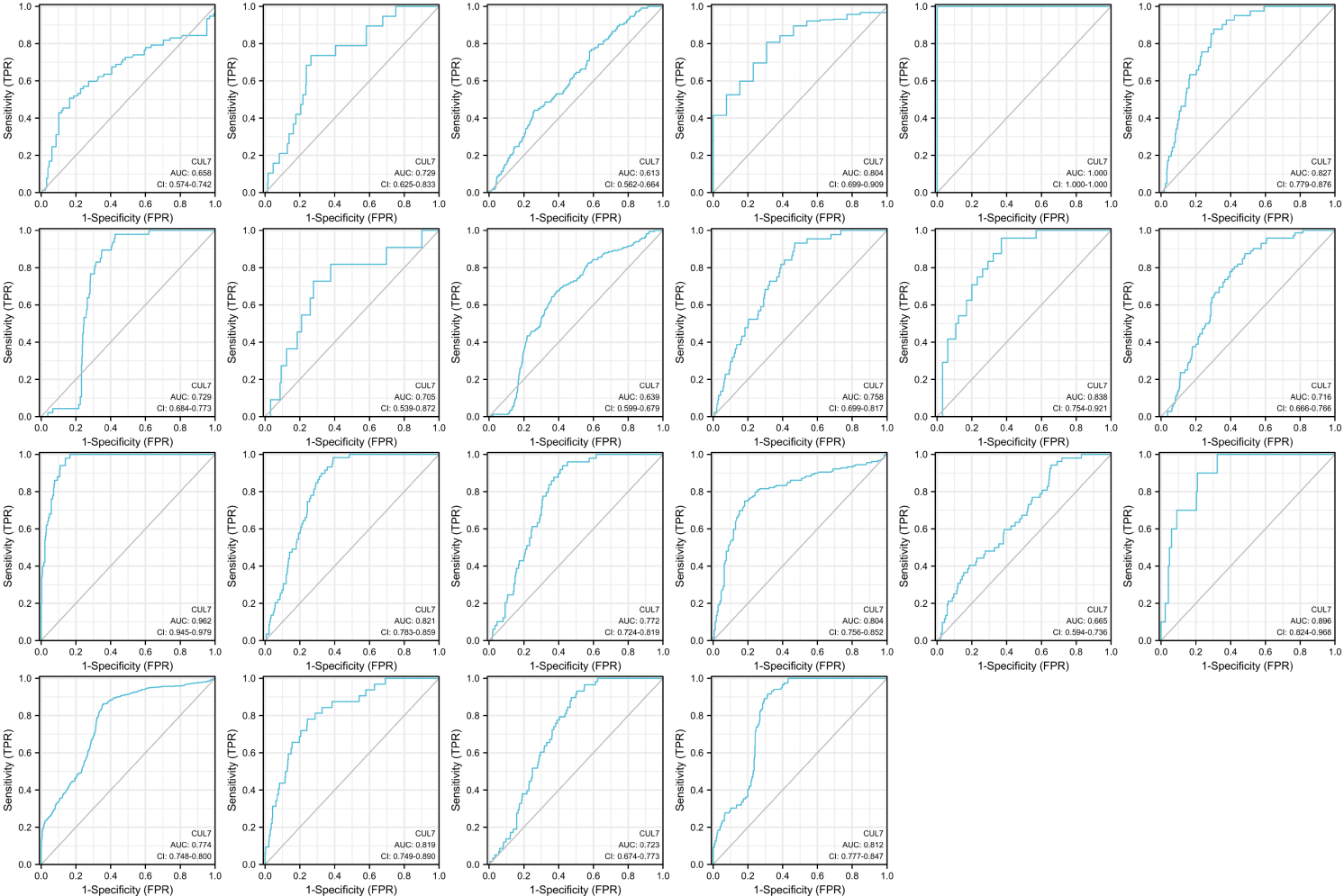


**Supplementary Figure 4.** The ROC curve of CUL7 expression and each tumor was used as a biomarker for diagnosis in tumor and normal tissues. AUC: area under the curve.


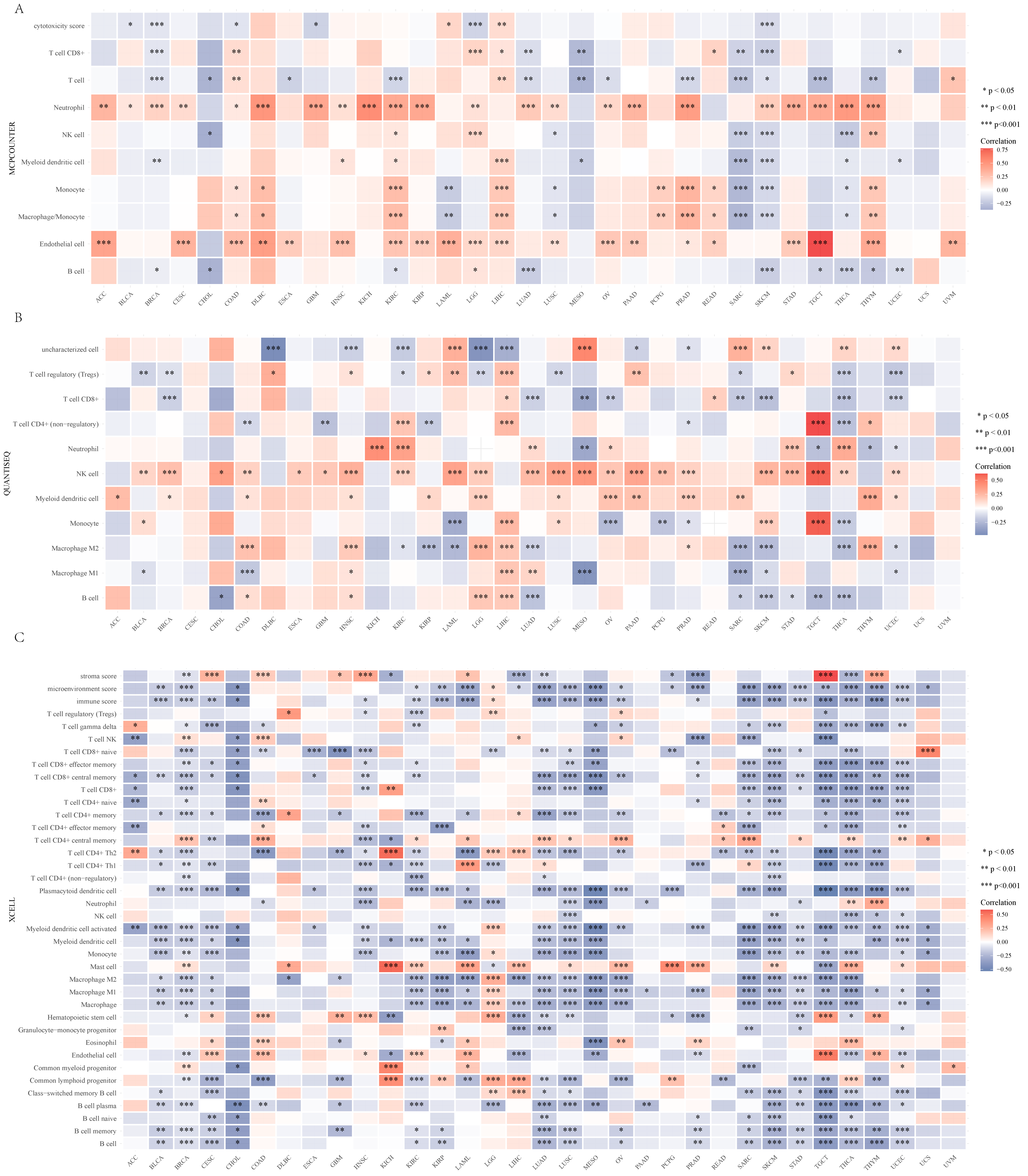


**Supplementary Figure 5.** Correlation Analysis between CUL7 expression and immune cell infiltration. (A) The correlation between CUL7 expression and immune cell infiltration was calculated according to MCPCOUNTER algorithm. (B) The correlation between CUL7 expression and immune cell infiltration was calculated according to the QUANTISEQ algorithm. (C) The correlation between CUL7 expression and immune cell infiltration was calculated according to xcell algorithm.


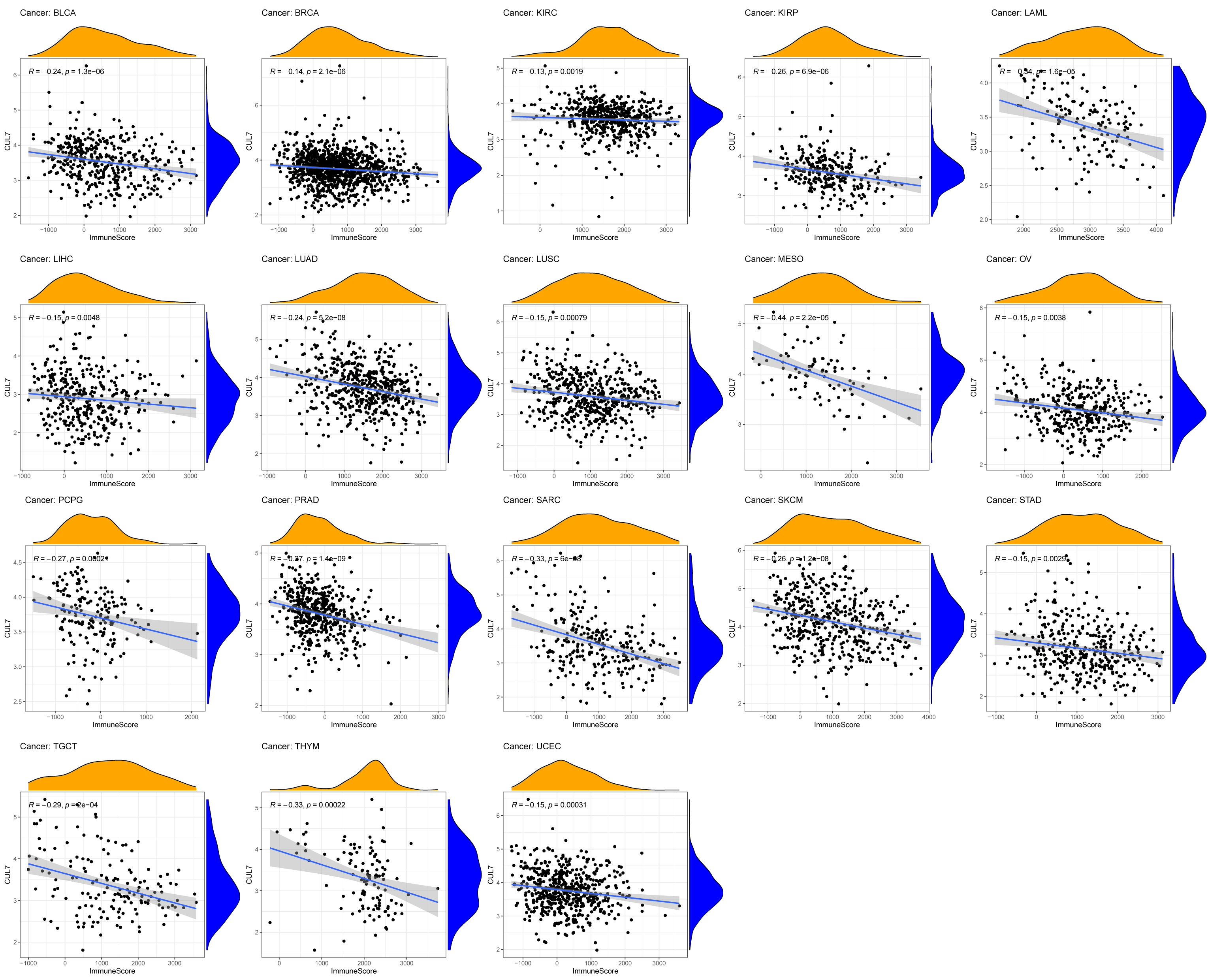


**Supplementary Figure 6.**Correlation Analysis between CUL7 expression and ImmuneScore.


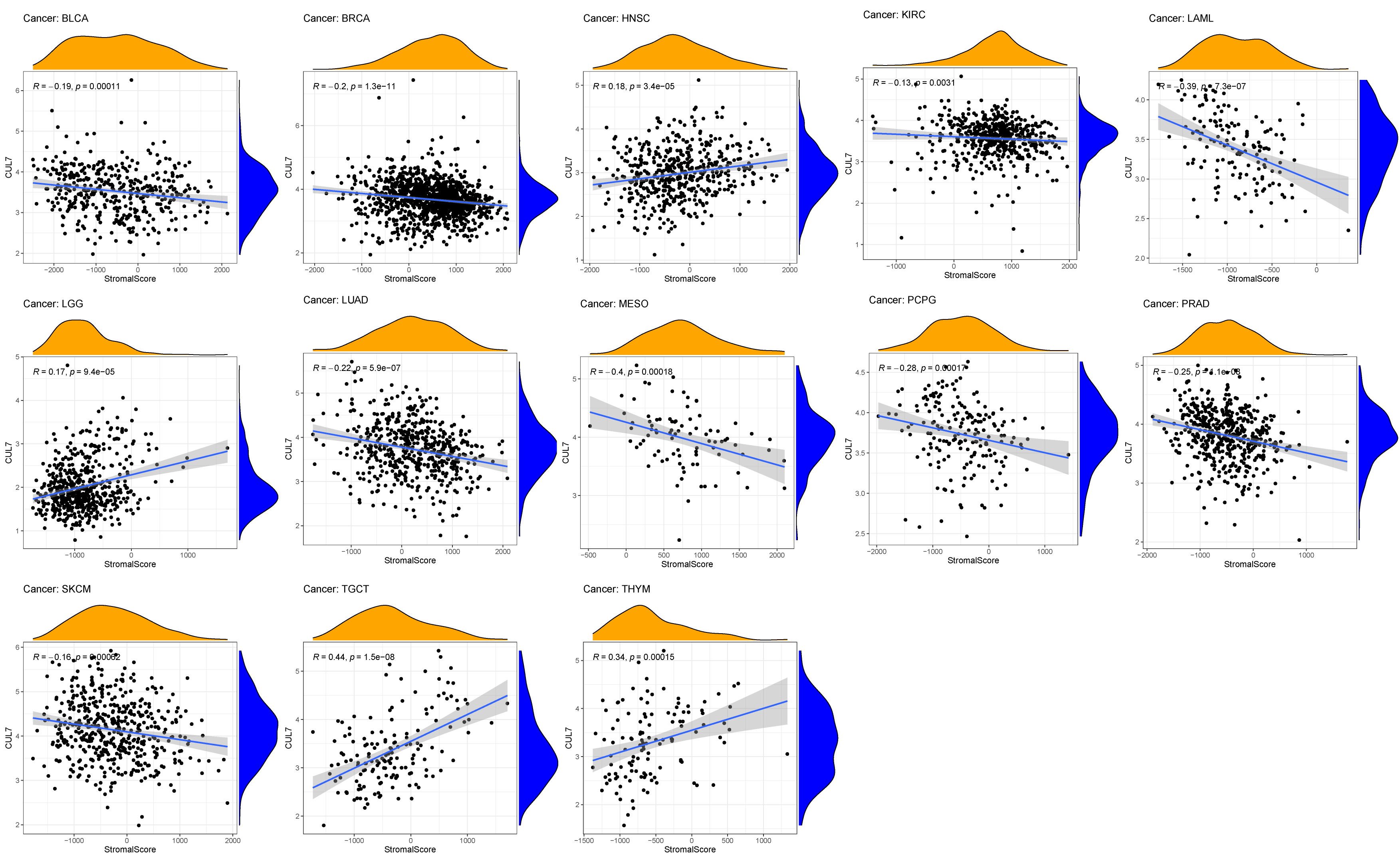


**Supplementary Figure 7.**Correlation Analysis between CUL7 expression and StromalScore.


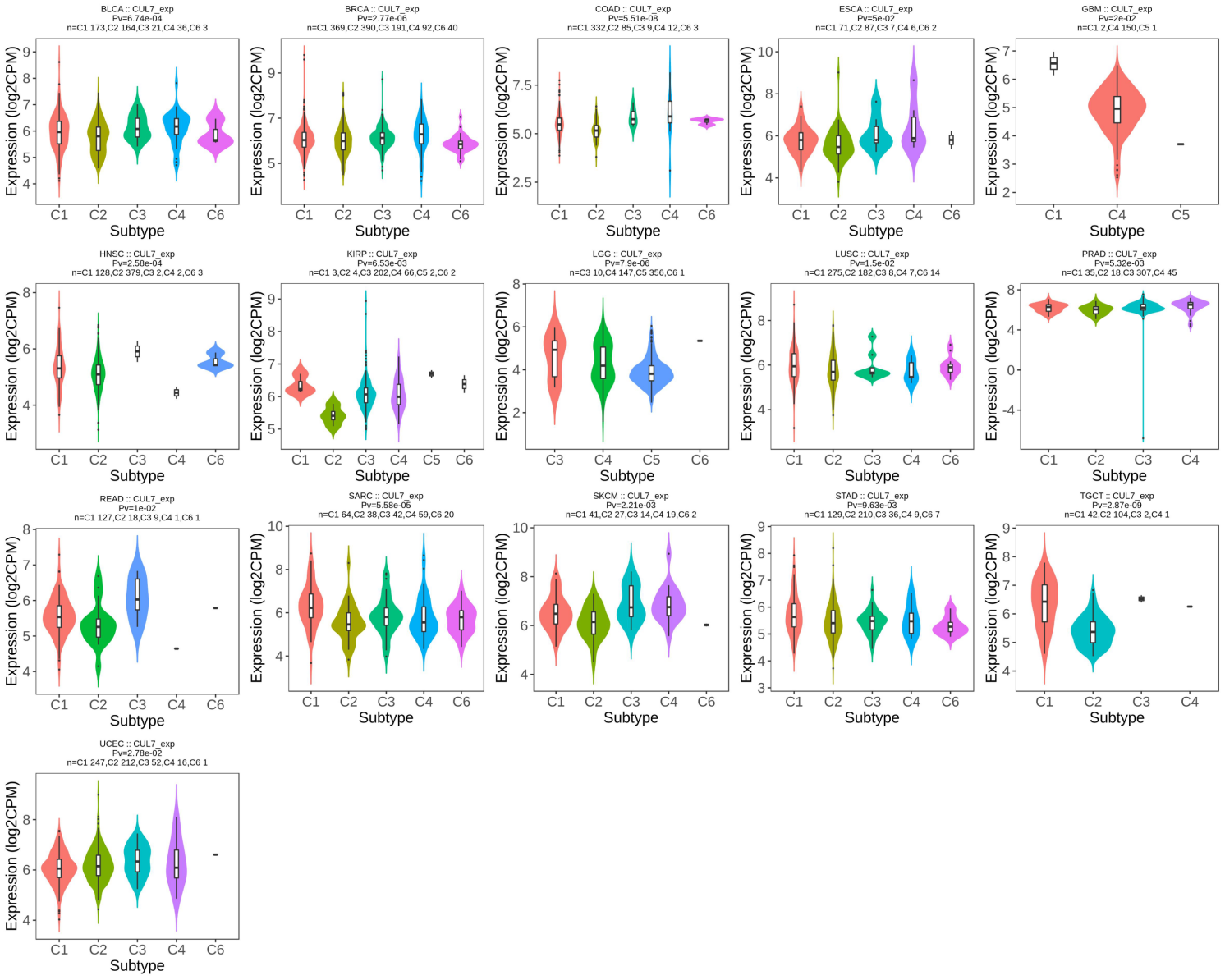


**Supplementary Figure 8.**Correlation between CUL7 expression and tumor immune subtypes. Expression of CUL7 in TISIDB in different tumor immune subtypes.


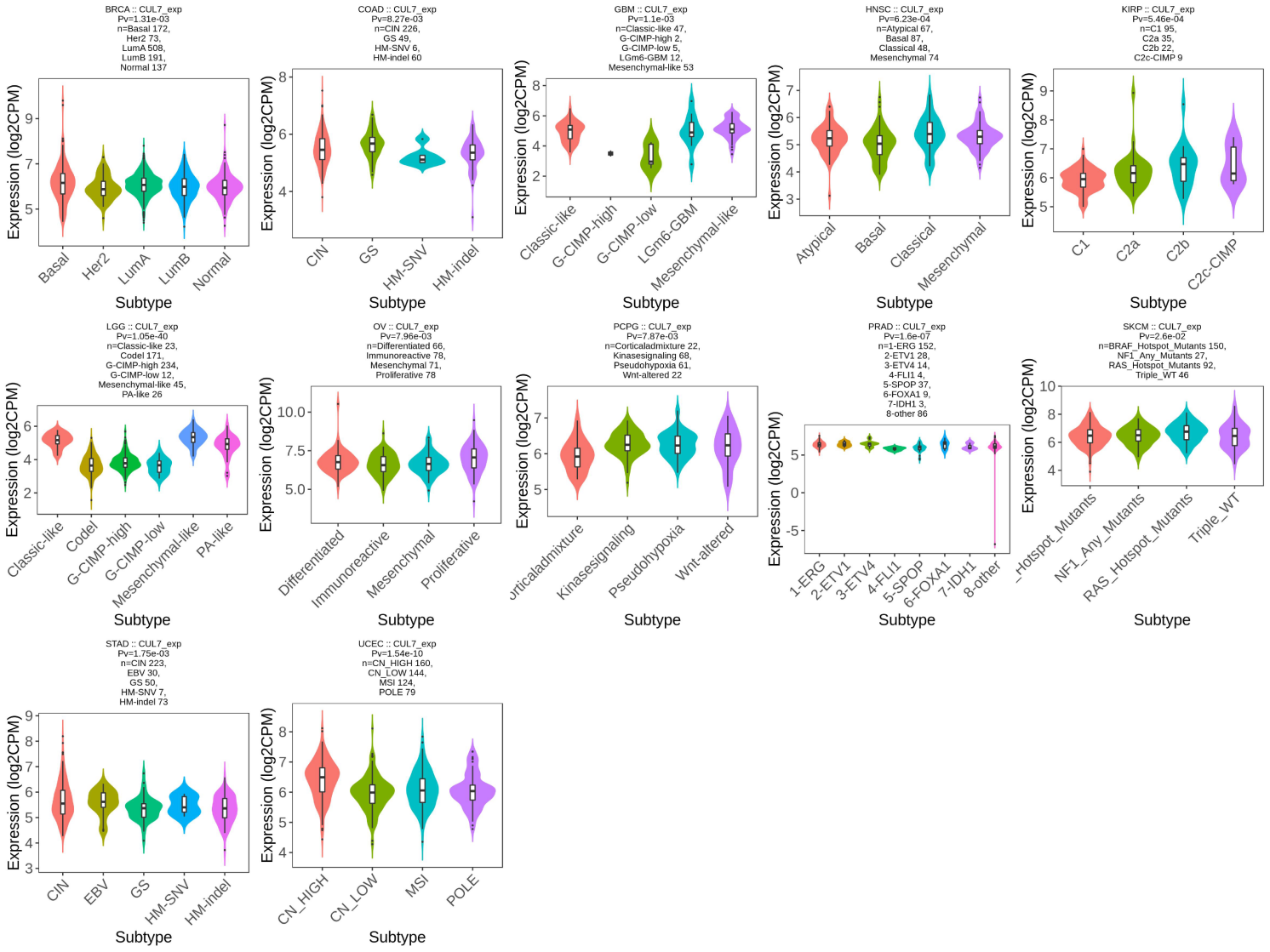


**Supplementary Figure 9.**Correlation between CUL7 expression and tumor immune molecules. Expression of CUL7 in TISIDB in different tumor molecular subtypes.


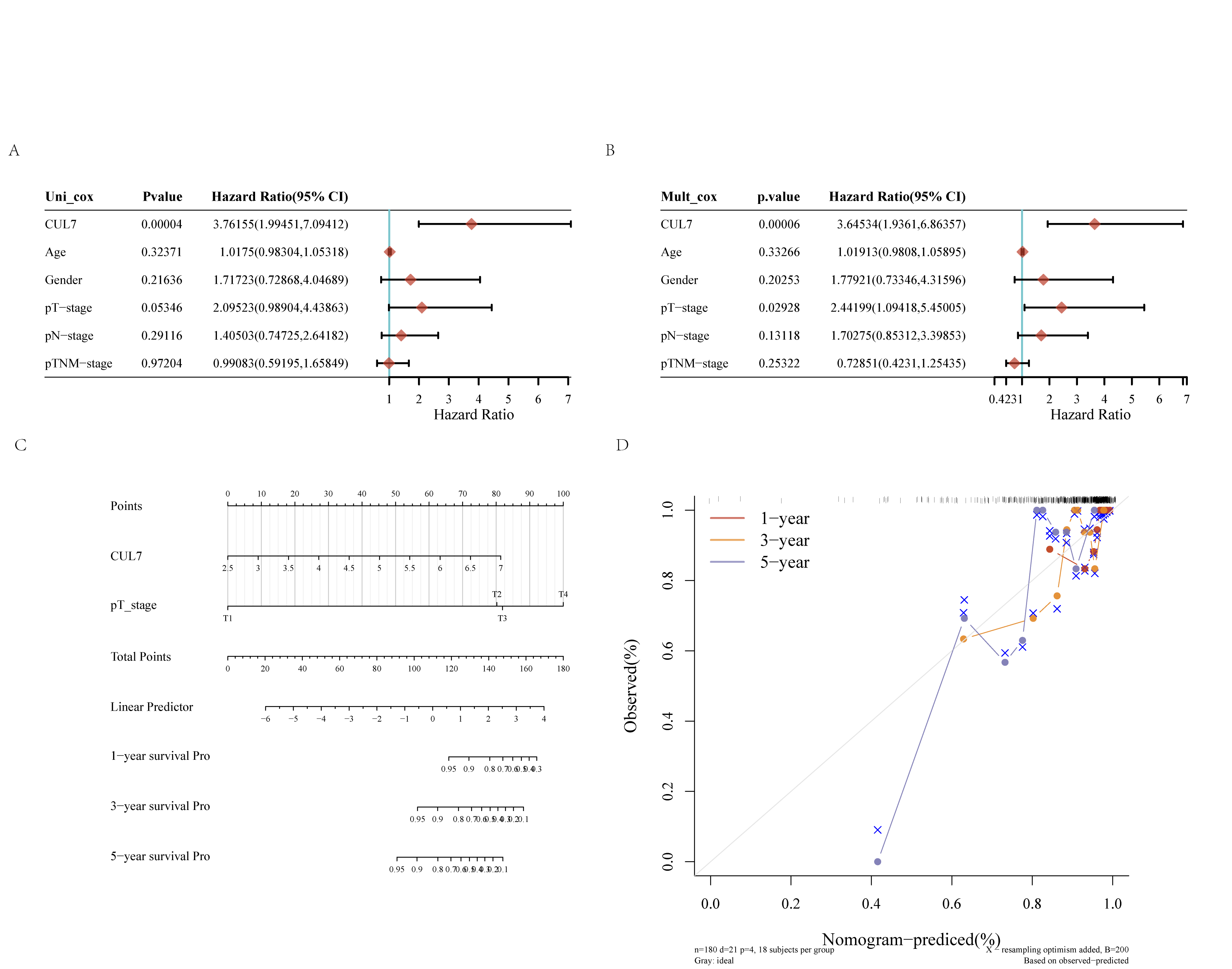


**Supplementary Figure 10.** Kaplan-Meier DFS analysis of COAD patients with high and low expression levels of CUL7.
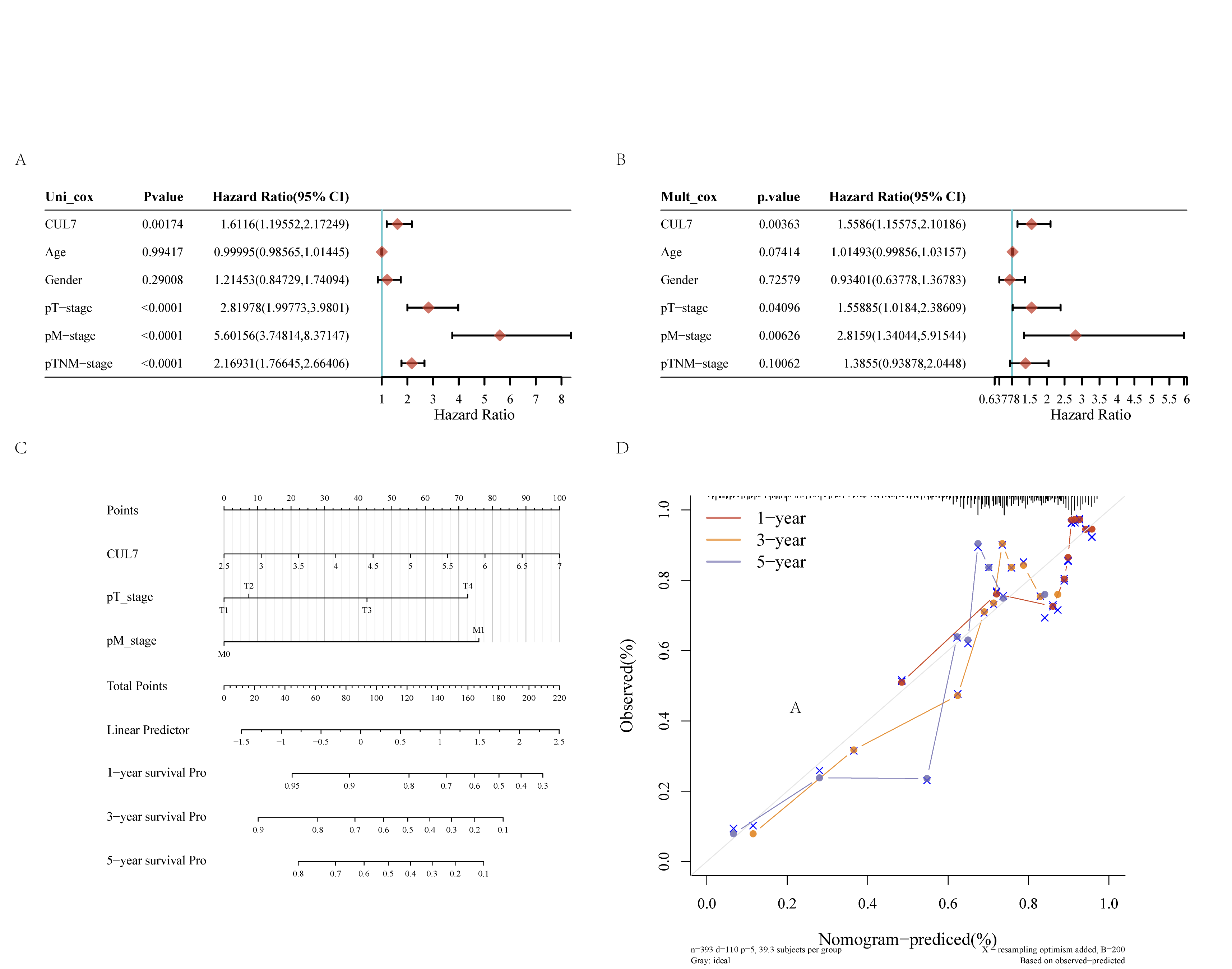


**Supplementary Figure 11.** Kaplan-Meier PFS analysis of COAD patients with high and low expression levels of CUL7.
